# Knowledge, attitudes and use of evidence-based practice among midwives in Belgium: A cross-sectional survey

**DOI:** 10.18332/ejm/147478

**Published:** 2022-06-10

**Authors:** Dorien Lanssens, Régine Goemaes, Christine Vrielinck, Inge Tency

**Affiliations:** 1 Scientific Research Working Group, Flemish Professional Organization of Midwives, Antwerp, Belgium; 2 Limburg Clinical Research Centre Mobile Health Unit, Faculty of Medicine and Life Sciences, Hasselt University, Hasselt, Belgium; 3 Department of Gynecology, Obstetrics and Fertility, Oost-Limburg Hospital, Genk, Belgium; 4 Az Damiaan General Hospital, Ostend, Belgium; 5 Department of Midwifery, Odisee University of Applied Sciences, Sint-Niklaas, Belgium

**Keywords:** attitudes, barriers, clinical practice guidelines, evidence-based practice, implementation, midwifery

## Abstract

**INTRODUCTION:**

Evidence-based practice (EBP) leads to improved health outcomes and reduces variability in the quality of care. However, literature on the knowledge, attitudes and use of EBP among midwives is scarce internationally and in Belgium.

**METHODS:**

A cross-sectional study using an online semi-structured questionnaire explored practice, attitudes and barriers on EBP and clinical practice guidelines. Midwives (n=251) working in university and non-university hospitals, primary care, and midwifery education, in Flanders (Belgium) were included.

**RESULTS:**

Midwives with a Master’s degree (57.7% vs 37.8%; p=0.004), ≤15 years since graduation (50.8% vs 35.5%; p=0.015) and aged <40 years (49.7% vs 34.6%; p=0.02), had better knowledge of the EBP-definition. The majority searched for literature (80.1%), mainly evidence-based (EB) clinical practice guidelines (50.6%), randomized controlled trials (45.0%) and systematic reviews (43.0%). Midwives found EBP necessary and realistic to apply in daily practice and support decision-making. They were willing to improve EBP-knowledge and skills but assumed to be competent in providing evidence-based care. Most respondents were convinced of the importance of EB clinical practice guidelines but did not believe guidelines facilitated their practices or enabled them to consider patient preferences adequately. Half of the midwives (55.8%) experienced barriers to EB clinical practice guideline use, mainly lack of time (35.9%), access (19.5%), and support (17.9%).

**CONCLUSIONS:**

Although midwives showed a positive attitude towards EBP, education programs to promote EBP and improve EBP-related knowledge and skills are needed. Future efforts should focus on developing strategies for overcoming barriers and enhancing the consistency of EBP implementation.

## INTRODUCTION

Since its introduction as evidence-based medicine in the late 1990s, the concept of evidence-based practice (EBP) has become fundamental in modern healthcare. Sacket et al.^1^ defined EBP as ‘the conscientious and judicious use of current best evidence in making decisions about the care of individual patients’. EBP should incorporate the best available and current research evidence, clinical expertise and judgment, and patients’ preferences (=EBP framework)^2^. The shift towards a multidisciplinary care approach emphasized the need to expand the evidence-based medicine framework to other health professions, including midwifery^3,4^.

The Belgian midwife is defined as an expert in pregnancy, childbirth and the maternity period. She is trained according to a three-year Bachelor’s degree in midwifery and may also have a two-year Master’s degree. The midwife profession is regulated under Belgian law and has a clear professional and competence profile. In addition, the midwife undergoes continuous training with 75 hours of constant training per 5 years. Therefore, she is medically trained and the expert *par excellence* to guide normal pregnancy and childbirth and the regular maternity bed^5^. In Flanders, 61700 births were registered in 2020, of which 99.30% happened in 59 hospitals, distributed over Flanders and Brussels (n=59). Births at the hospitals mainly occur under the supervision of an obstetrician. The midwife guides and supports the women during labor and delivery; 0.7% (n=406) of the Flemish births happen at home, under the midwife’s supervision^6^.

The research-driven character of the midwifery profession has also been reflected in the Belgian competency profile, which stipulates that ‘the midwife provides evidence-based care by integrating new scientific insights into practice. She conducts and participates in scientific research’^5^. This aligns with the research-driven character in several competency profiles across Western countries. EBP has become an obligatory element in healthcare systems and regulations due to the demand for high quality, and at the same time, cost-effective care. Childbearing women and their families can benefit from evidence-based midwifery practice, as it leads to improved health outcomes and reduces the variability in quality and provision of care. The structured process facilitates transparent decision making^7-11^. Policymakers are sensitive to this reality. Therefore, the importance of EBP in many governmental initiatives has been emphasised^3-4^. Despite several efforts and investments by national policies, midwives struggle to incorporate EBP in perinatal care^9,12,13^. Only a few studies have been executed to explore midwives’ knowledge and attitudes towards EBP. Midwives who participated in these surveys reported EBP as an essential mindset in clinical practice, but only a very small percentage practiced within an EBP framework. Many lacked the knowledge, skills and confidence to implement it effectively instead of relying on intuition and personal clinical experience^2,8,10^. Earlier studies have addressed several barriers, mainly conducted among nurses^10^. Inhibiting factors are at the individual level of the caregiver and the organizational level, which suggests that both top-down and bottom-up approaches are mandatory for EBP. Lack of time, resources and skills have been marked as critical barriers. A shortage of staff, lack of autonomy and lack of interest from supervisors have also been reported in qualitative research^9,11^.

A bottom-up strategy is mandatory to overcome these barriers. The empowerment of midwives’ research skills and the enforcement of a positive attitude towards EBP are critical factors for a successful approach^9-11,14,15^. Mohammadi et al.^14^ tested the ‘diffusion of innovation theory’ in a model for EBP adoption among nurses. The results showed a strong positive correlation of knowledge and attitudes with EBP adoption. Unfortunately, data regarding these factors and EBP among midwives remain sparse. Most current studies are qualitative, exploratory, or analyzed data of nurses and midwives together^2,9,13^. One Iranian study found a significant difference in EBP knowledge and attitudes between nurses and midwives^8^. These findings illustrate the relevance of separate data-analysis of EBP-related beliefs and skills for midwives, as these are influenced by occupational culture and contextual factors^3,12^.

In Belgium, the Ministry of Health introduced a national policy regarding disseminating and implementing EBP, with evidence-based (EB) clinical practice guidelines playing a pivotal role. Hence, the Belgian Health Care Knowledge Centre (KCE) surveyed Belgian healthcare professionals to assess their perceptions, needs and general use of EB clinical practice guidelines^4^. However, the use of EB Clinical Practice Guidelines depicts a particular component of evidence-based practice and does not cover the complete process of EBP. Therefore, a more extensive EBP-survey was developed for the present study, focusing on midwives working in Flanders, the Dutch-speaking northern part of Belgium.

## METHODS

### Study design and population

A cross-sectional online survey using a semi-structured, self-administered questionnaire was conducted in Flanders from May to October 2017. A total of 251 midwives working in university and non-university hospitals, primary care, and midwifery education were recruited through electronic newsletters, social media and the journal of the Flemish Midwives Association. Posters with study information were also distributed on labor and maternity wards of all Flemish hospitals and one Dutch-speaking hospital in the Brussels-Capital Region.

### Data collection

The questionnaire was developed after a literature review and was partially based on the questionnaires of similar studies among physical therapists^15-17^. Psychometrics were done in those studies, and the questionnaires were judged to have robust validity and internal reliability. The questions were then adapted to the professional practice of midwives in Flanders. Subsequently, an online two-round Delphi study with a panel of 10 experts was undertaken to evaluate the questionnaire’s content validity and assess its items’ readability, clarity, and comprehensibility. The experts were lecturers in midwifery, (head) midwives, an obstetrician, and experts from KCE regarding implementing an integrated, evidence-based practice plan in Belgium. They rated the questions and items using a 6-point Likert scale (1=strongly disagree, to 6=strongly agree). Questions and items were considered valid when consensus was reached, and a minimum median score of 4 was obtained. No questions or items were deleted after the first round. However, one sociodemographic variable regarding position appointment percentage was added, and five items were reworded based on the experts’ comments. An adapted version of the questionnaire was assessed in a second round, after which consensus was reached on all questions and items. The final version of the questionnaire comprised four parts that evaluated: 1) sociodemographic characteristics (12 multiple choice questions); 2) knowledge and practices (4 multiple choice questions); 3) attitudes, advantages and disadvantages regarding EBP (9 propositions rated on a 5-point Likert scale from strongly disagree to agree strongly); and 4) attitudes towards the use of clinical practice guidelines. The latter part contained five multiple-choice questions, one open question, and nine propositions rated on a 5-point Likert scale. The questionnaire was semi-structured as it included a combination of the multiple-choice questions and propositions rated on a 5-point Likert scale (where predefined answers were given) and an open question (where the midwives could provide their opinion).

### Statistical analysis

Data were analyzed using IBM SPSS Statistics 24.0 software. The analysis mainly focused on descriptive statistics. Chi-squared tests were applied to identify differences in categorical variables. Results with p<0.05 were considered statistically significant.

## RESULTS

### Characteristics of the participants

In total, 256 questionnaires were completed and returned. Of these, 251 were included in the final analysis. Five questionnaires were excluded because participants were not midwives (n=3), or confirmation of a Bachelor’s degree in midwifery was missing (n=2). Participants were geographically spread over Flanders, varying from 11% to 29% per region (Supplementary file). The majority of the respondents had more than ten years of working experience (64.2%) and were younger than 40 years (58.6%). The median number of years after graduating as a midwife was 15 [(IQR: 8–27). The education level of half of the participants was a Bachelor’s degree (53.8%). Most midwives worked in clinical practice, either in a hospital (37.8%), primary care (25.5%) or both (26.3%). The majority of the respondents had a computer or tablet at home (99.6%). All midwives had Internet access at home and work. Characteristics of the study participants are summarized in Table 1.

**Table 1:** Characteristics of the study participants (N=251)

*Variables*	*n (%)*
**Age** (years)	
<40	147 (58.6)
≥40	104 (41.4)
**Educational level**	
Bachelor’s	176 (70.1)
Master’s	71 (28.3)
PhD	4 (1.6)
**Work experience as a midwife** (years)	
≤10	110 (43.8)
>10	141 (56.2)
**Years since graduation**	
≤15	130 (51.8)
>15	121 (48.2)
**Employment percentage as a midwife**	
<50	49 (19.5)
≥50	202 (80.5)
Place of employment*	
Hospital	95 (37.8)
Primary healthcare	64 (25.5)
Primary healthcare in combination with a hospital	66 (26.3)
Higher education	54 (21.5)
Bachelor’s education	42 (77.78)
Master’s education	8 (15.38)
Other	4 (7.41)
**Type of hospital** (n=161)	
Non-university hospital	118 (73.3)
University hospital	43 (26.7)

* Midwives may have several places of employment.

### Knowledge of EBP

Evidence-based practice was part of the Bachelor’s curriculum for 55.4% of the participants, and 27.1% reported that they had followed education or training on EBP. Four in ten respondents were aware of the definition of EBP and answered correctly to the question on ‘how to define EBP’ (43.4%) (Table 2).

**Table 2:** Knowledge regarding EBP definition

*What do you understand of the term EBP?*	*n (%)*
The use of scientific literature is the basis for daily practice.	75 (23.9)
Scientific literature in combination with the preference of the client is the basis for daily practice.	13 (5.2)
Scientific literature, practical experiences of the professional and the preference of the client is the basis for daily practice.	109 (43.4)
Practical experiences in combination with the preferences of the client is the basis for daily practice.	2 (0.8)
Practical experiences in combination with the scientific literature is the basis for daily practice.	52 (20.7)

A significant association was found between knowledge of the definition of EBP and the variables: educational level, age, and years since graduation. Midwives with a Master’s degree were more likely to identify the correct definition of EBP than midwives with a Bachelor’s degree (57.7% vs 37.8%, χ²=8.264, p=0.004). Also, midwives aged <40 years and midwives with ≤15 years since graduation were more able to define EBP correctly (49.7% vs 34.6%, χ²=5.611, p=0.02; and 50.8% vs 35.5%, χ²=5.918; p=0.02, respectively). There was no significant correlation between knowledge of the EBP definition and the percentage of employment, EBP as part of the Bachelor’s curriculum, type of hospital, or working experience as a midwife.

### Attitude towards EBP and EB clinical practice guidelines

As indicated in Table 3, most midwives were convinced that implementing EBP in daily practice is necessary (85.2%) and realistic (59.3%). Most midwives also agreed to learn (73.3%) and improve (82.5%) knowledge and skills needed to apply EBP. In all, 66.1% believed that there is sufficient scientific evidence for most of their care, and 80.9% thought that EBP could support decision-making during the care they deliver. Half of the midwives were stimulated by their working environment to use scientific literature (50.6%) and reported sufficient skills to search for relevant literature (51.4%). Two-thirds were convinced about their abilities to deliver care following the most recent evidence (59.4%).

**Table 3:** Attitude of midwives regarding EBP and EB clinical practice guidelines

*Statement*	*Completely disagree n (%)*	*Not agree n (%)*	*Neutral n (%)*	*Agree n (%)*	*Totally agree n (%)*
I think it is necessary to implement EBP in my daily practice	1 (0.4)	5 (2.0)	31 (12.4)	109 (43.4)	105 (41.8)
I think it is realistic to implement EBP in my daily practice	3 (1.2)	23 (9.2)	76 (30.3)	114 (45.4)	35 (13.9)
I want to acquire the knowledge and skills necessary to implement EBP in my daily practice	2 (0.8)	12 (4.8)	53 (21.1)	107 (42.6)	77 (30.7)
I want to improve the knowledge and skills necessary to implement EBP in my daily practice	2 (0.8)	8 (3.2)	34 (13.5)	119 (47.4)	88 (35.1)
There is a lack of strong scientific evidence for most actions/care that I carry out	42 (16.7)	124 (49.4)	55 (21.9)	24 (9.6)	6 (2.4)
EBP can support me to make decisions towards care	3 (1.2)	8 (3.2)	37 (14.7)	152 (60.6)	51 (20.3)
The use of scientific literature is encouraged in my working environment	16 (6.4)	50 (19.9)	58 (23.1)	74 (29.5)	53 (21.1)
I have sufficient skills to search for relevant scientific literature	11 (4.4)	37 (14.7)	74 (29.5)	83 (33.1)	46 (18.3)
I am convinced of my skills to deliver care following the most recent evidence	2 (0.8)	15 (6.0)	85 (33.9)	119 (47.4)	30 (12.0)
It is important to have easy access to EB clinical practice guidelines (i.e. costless, electronically and quickly available)	0 (0)	5 (2.0)	16 (6.4)	63 (25.1)	167 (66.5)
It is important to use EB clinical practice guidelines during my work	1 (0.4)	9 (3.6)	24 (9.6)	125 (49.8)	92 (36.7)
I have easy access to relevant EB clinical practice guidelines in my work place/through my employer	24 (9.6)	63 (25.1)	56 (22.3)	72 (28.7)	36 (14.3)
I have easy access to EB clinical practice guidelines at home	20 (8.0)	56 (22.3)	77 (30.7)	70 (27.9)	28 (11.2)
By using EB clinical practice guidelines, client preferences can be sufficiently taken into account	5 (2.0)	33 (13.1)	102 (40.6)	100 (39.8)	11 (4.4)
EB clinical practice guidelines are necessary to facilitate care	7 (2.8)	28 (11.2)	85 (33.9)	114 (45.4)	17 (6.8)
EB clinical practice guidelines are important in order to give clients high-quality care	4 (1.6)	6 (2.4)	25 (10.0)	127 (50.6)	89 (35.5)
EB clinical practice guidelines are important in order to give clients equivalent care	5 (2.0)	13 (5.2)	42 (16.7)	136 (54.2)	55 (21.9)
I use EB clinical guidelines in my daily practice	4 (1.6)	17 (6.8)	69 (27.5)	115 (45.8)	46 (18.3)

The majority of the midwives found easy access to EB clinical practical guidelines crucial (91.6%) and the use of guidelines during their work essential (86.5%). However, only 43.0% of them mentioned having easy access to relevant EB clinical practice guidelines at their work and 39.1% at home. Almost one-third of the respondents (30.7%) responded neutrally towards the accessible access facility toward guidelines at home. Although 44.2% agreed that the client’s preferences could be sufficiently taken into account when using EB clinical practice guidelines, 40.6% were neutral about this statement. The same answer was obtained when asked about the facilitating role of guidelines in delivering care (52.2% agreed, 33.9% neutral). Most midwives agreed that EB clinical practice guidelines are essential in giving high quality (86.1%) and equal (76.1%) care to clients. Finally, 64.1% of the midwives agreed that they use guidelines in their work, while one-third were neutral (27.5%).

A significant association was found between searching for scientific literature and educational level and working experience variables. A significantly higher proportion of midwives with a Master’s degree searched for scientific literature than midwives with a Bachelor’s degree (95.8% vs 73.9%, χ²=12.288, p<0.001). Furthermore, a higher number of midwives with >10 years of work experience were seeking more scientific literature than midwives with ≤10 years of experience (86.4% vs 75.2%, χ²=4.847, p=0.03). No significant correlation was seen between searching for literature and the percentage employed, EBP as part of the Bachelor’s curriculum, type of hospital, age or years since graduation.

### Practice towards EBP and EB clinical practice guidelines

The majority of the respondents (80.1%) searched for scientific literature, especially for EB clinical practice guidelines (50.6%), randomized controlled trials (RCTs) (45.0%) and systematic reviews (43.0%) (Table 4). They searched for scientific literature mainly by using PubMed (58.6%) and search machines on the Internet (e.g. Google) (43.0%). Midwives read one (40.6%) or two to five (31.9%) scientific publications every month. Most midwives were aware of relevant existing EB clinical practice guidelines (73.7%) and informed where to find them (71.3%). However, only half of them knew how to search for guidelines on the Internet (55.8%).

**Table 4:** Practice towards EBP and EB clinical practice guidelines among midwives (N=251)

*Question*	*n (%)*
**What kind of scientific literature do you search for?**	
Systematic reviews	108 (43.0)
Meta-analyses	61 (24.3)
Observational studies	31 (12.4)
RCTs	113 (45.0)
Case studies/case reports	61 (24.3)
EBP guidelines	127 (50.6)
Other	16 (6.4)
**How do you get access to scientific literature?**	
By a database made available by the employer	49 (19.5)
By CEBAM*	54 (21.5)
By a search engine on the Internet (e.g. Google)	108 (43.0)
By PubMed	147 (58.6)
By Web of Science	37 (14.7)
By another source	27 (10.8)

* CEBAM: Belgian Centre for evidence-based medicine Cochrane Belgium.

### Barriers toward EB clinical practice guidelines

**Figure 1:**
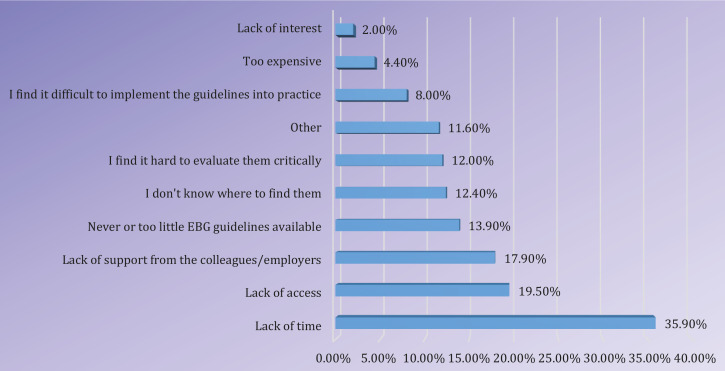
Barriers towards the use of EB clinical practice guidelines

As shown in Figure 1, the majority of the participants mentioned lack of time (35.9%) as one of the most important impediments for the use of EB clinical practice guidelines, followed by lack of access (19.5%) and lack of support from colleagues or employers (17.9%).

There was no significant correlation between whether midwives experienced barriers for using EB clinical practice guidelines and the variables employment, EBP as part of the Bachelor’s curriculum, type of hospital, educational level, age, working experience as midwife or years since graduation as a midwife. However, a lower number of midwives working in a university hospital experienced barriers than midwives working in a regional hospital (44.2% vs 61.9%, χ²=4.022, p=0.05) as well as participants with >10 years of work experience as midwives compared to those with ≤10 years (50.4% vs 62.7%, χ²=3835, p=0.05).

## DISCUSSION

### Current knowledge and attitude towards EBP

This study indicates that midwives show a positive attitude towards EBP. However, education programs to promote EBP and improve EBP-related knowledge and skills are still needed. This need is reflected in the study results, as 82.5% of the respondents wanted to improve their knowledge and skills to implement EBP in their daily work. But only half of the midwives (51.4%) were convinced about their skills to search for relevant literature. These results are consistent with previous studies describing attitudes, practices and knowledge/skills associated with EBP^18^. Therefore, midwives’ EBP attitudes commonly differed from their ability to implement EBP, which is not a unique finding for only midwives in Flanders.

This study also indicates that midwives with the best knowledge of the definition of EBP were the midwives with a Master’s degree, who had ≤15 years since graduation and were aged <40 years; this may be because these midwives still knew the definition of EBP from their midwifery education or still used the skills they had learned to search after EBP. Older midwives or midwives with longer work experience need to review updated guidance due to time elapsed since graduation. The majority of the midwives searched for literature, mainly EB clinical practice guidelines, RCTs and systematic reviews. The academic degree made a difference in the search for scientific literature, as midwives holding a Master’s degree reported a higher rate of searching for evidence-based information than respondents with a Bachelor’s degree. These results are consistent with those reported in previous studies which found that nurses with Master’s degrees reported fewer barriers to finding and reviewing research than nurses with Bachelor’s degrees^8,19^. An explanation could be that a Master’s education program prepares its graduates to read and understand research findings. At the same time, this is not always the case in Belgium’s Bachelor’s program in midwifery. Majid et al.^20^ concluded that creating an environment that provides opportunities for caregivers to share knowledge and information should be a key priority for hospital management.

Lastly, the reported results indicate that midwives found EBP necessary, realistic to apply in daily practice, and supported decision-making. Most respondents were convinced of the importance of EB clinical practice guidelines but did not believe these facilitated their training or enabled them to consider patient preferences adequately.

### Facilitators and barriers to research utilization

Findings from the present study mirror the facilitators and barriers to research utilization experienced by nurses and midwives in other countries. A positive attitude towards EBP was found to be a facilitator for the use of EBP. Over half of respondents (54.2%) were convinced of the importance of EB clinical guidelines for high-quality midwifery care for normal pregnancy, birth and the postnatal period. They did believe policies facilitate their caregiving and enable them to consider patient preferences adequately (50.6%). This means that the other half of the respondents were neutral or did not agree that guidelines support taking client preferences into account or facilitating caregiving. Additional research is needed to understand midwives’ beliefs regarding this aspect of our findings.

The top-three ranking of barriers for guideline use among Flemish midwives in our study were: 1) lack of time (35.9%), 2) lack of access (19.5%), and 3) lack of support (17.9%). These results reflect the findings among nurses and midwives. The most cited barriers to nurses’ use of research were primarily based on: 1) lack of time to practice database searching in a hectic work environment, 2) lack of access to databases, 3) lack of skills and resources, 4) inadequate organizational support, 5) not readily available results of research, and 6) delayed publication of research reports and articles^11,18,19,21^. Cummings et al.^22^ thought that a better insight into the practical environment is crucial to the understanding and developing interventions advancing EBP in the midwives’ community. In this light, hospital management should consider adjusting midwives’ work schedules for them to have additional time to attend classes on conducting EBP, reviewing relevant literature, and planning functional changes.

### Scientific literature consultation by Flemish midwives

The current study measured the frequency of scientific literature consultation by midwives. Just over half (54.4%) either did not read articles or read only one per month. However, 85.2% of the respondents believed it is necessary to implement EBP in their daily practice. These findings illustrate the gap between EBP-related attitudes and the actual procedure. Since 64.1% of the participants were working in a hospital setting, a possible explanation for this finding could be that several clinical guidelines were available for these midwives. One or more midwives developed these clinical guidelines in collaboration with the obstetrician and pediatricians, and were based on the current best evidence. Therefore, the midwives feel no need to read articles and improve their EBP skills. However, this should still be recommended. This is even though most midwives working at hospitals have access to computers and scientific literature via the hospital where they work. Another possible reason why the respondents did not read as many articles, although they believed in the necessity to implement EBP in daily practice, could be that the physicians mostly decided on the care given to women. Only 0.7% of the Flemish mothers chose to give birth at home^6^. This means that most births in Flanders take place in a hospital setting, wherein obstetricians are the responsible actors taking the decisions during pregnancy, labor, delivery, and postpartum. The midwife supports women during pre-, peri- and postnatal care but does not act autonomously in hospitals. This dynamic might be reflected in the response rate. The midwives in this study seemed to be less interested in EBP because of their limited involvement in hospital decision-making.

Although the organization of midwifery in Belgium is somewhat unique compared to other countries in Europe (where midwives have a more autonomous function in supporting women throughout pregnancy, labor and conducting births on the midwife’s responsibility), the conclusions of this study can be generalized over Europe^23^. Our findings are in line with those of Ladopoulou et al.^24^ and Cleary-Holdforth^25^: for a successful implementation of EBP, it is required initially to train personnel to develop their abilities, to provide information on the way to use different data sources and encourage midwifery personnel to take initiatives and be part of the decision-making process. Additionally, midwifery across the world is facing changes and uncertainties. Within midwifery, different paradigms are embodied in the medical and biopsychosocial models, allowing us to consider technocratic, medicalized, and interventionist birth versus physiological birth, focusing on maternal emotional well-being and maternal and family life balance. Medicalization and the medical hierarchy are likely to influence midwives’ job conditions as technocratic and interventionist birth affects midwives’ ability to provide independent practice and their advocacy for physiological birth. Without a doubt, all of these factors impact the future of the midwifery profession, the organization of midwifery care, and the education of future midwives^26^. When further outlining the future of midwifery in Belgium (and therefore associated midwifery organization and education), it is essential to consider the results of this research.

### Strengths and limitations

This study was the first to evaluate Flemish midwives’ knowledge, attitude, and practices regarding EBP. In the present study, all participants were distributed equally among the different geographical regions, and midwives from various working fields participated. The respondents were active in a hospital setting, primary care, or education. Although this study used a semi-structured self-administered questionnaire, several measures were taken to guarantee the validity of the questionnaire. The response bias in this study was countered by formulating an equal number of questions generating positive and negative responses. In addition, extensive literature research was conducted to ensure the content validity of each item in the knowledge part. The questionnaire was also validated among a panel of experts.

There are some limitations to consider. The knowledge findings can be slightly biased due to the consultation of external sources (e.g. the Internet, colleagues). Owing to the cross-sectional design of the present study, only associations and no causations can be inferred. The response rate was lower than the desired rate of 65%^27^. Consequently, the findings may not represent the total population of midwives. The moderate individual response rate may have resulted from a concurrence of several factors, including a high workload of midwives or being less interested in the subject. The recruitment method relied on voluntary response sampling and may not truly represent the midwifery population in Flanders. Lastly, there is a risk for selection bias as midwives with a high level of interest was possibly more motivated to respond and undertook more effort to complete the questionnaire^15^. This high level of interest can explain the high rate of Master’s degree holders who participated in this study. Therefore, the scores might have been biased in favor of EBP. Nevertheless, we believe that the findings might provide valuable insights for optimizing midwives’ knowledge, attitudes, and practice among EBP.

### Recommendations for further research

To improve quality of care, it is necessary to create an EBP culture that investigates the barriers to EBP use, and that facilitates the implementation of the best evidence for pregnant and/or delivering women based on their preferences and values. Nowadays, there are only limited Flemish national clinical guidelines for a particular aspect of antenatal or perinatal care, which we can find (for instance) on https://ebpnet.be/. Policies and procedures for seeking, verifying, and aligning the best and current evidence should be standardized and integrated across the healthcare system. Written EB clinical guidelines should be readily accessible to midwives regardless of the working environment and across the healthcare system in order to achieve a successful and sustainable implementation of EBP in midwifery practice daily. Also, EB clinical guidelines should be formulated in collaboration between obstetricians and midwives. This would incorporate the needs of midwives that consider themselves both ‘autonomous’ and working in partnership with a lead obstetrician.

Our study showed a relation between the demographics of the midwives (age, work experience, educational level) and the attitudes, barriers and practice towards EBP, but, as far as we know, nothing is known about the possible links for this association. Additional research would be interesting to deepen this relation, and the other factors we identified in relation to the implementation of EBP, so we know how to reach every midwife (irrespective of the demographic background) to implement EBP guidelines. Understanding midwives’ use of best available evidence in practice will direct efforts towards developing mechanisms that facilitate the timely uptake of the latest evidence by all maternity care providers working in clinical settings^28^.

Since only 37.8% of the Bachelor’s and 57.7% of the Master’s degree holders indicated the correct definition of EBP, there is still an essential role for education to teach EBP. This is in line with previous studies, indicating a lack of knowledge among caregivers who rely on intuition and personal clinical experience^2,8,10^. The survey of Mohammadi et al.^14^ showed a strong positive correlation between knowledge and attitudes of EBP. To improve attitudes towards EBP, caregivers must understand the true meaning of EBP, which unites the current best evidence with the patients’ preferences and the caregiver’s expertise.

There are several accreditation programs in Flanders to improve the quality of care and patient safety in hospitals. These accreditation programs can be used to implement EBP in multidisciplinary teams. Recent publications also highlight the possibility to implement a web-based resource that standardizes the process of evidence implementation. Midwives need practical solutions and a map of the process to lead implementation of evidence-based practices, packaged into a centralized web-based resource^29^. Together with midwifery associations and organizations promoting EBP, the Ministry of Health can improve access to databases and provide time for caregivers to search and read scientific literature. To enhance the EBP skills of midwives, they need the opportunity to read scientific literature. There is a vital role for the educational institutions to teach future midwives the meaning and importance of EBP and offer lifelong learning courses to maintain their knowledge, attitudes and practice towards EBP.

## CONCLUSIONS

The majority of participants had a positive attitude towards EPB. However, less than half of the midwives correctly defined EPB. Knowledge of EBP was associated with education level, age, and years since graduation. Hence, education programs are needed to promote EBP and improve midwives’ skills and knowledge, especially for midwives without a Master’s level education and midwives who graduated more than 15 years ago. Results also showed that a minority of participants had easy access to EB clinical practice guidelines both at their workplace and home, despite many searching for scientific literature. It is necessary to elaborate strategies to overcome barriers to access scientific literature. Additionally, enhancing the quality and consistency of EBP implementation is required.

## Data Availability

The data supporting this research cannot be made available for privacy reasons.
